# F133. ARE WE UNDERESTIMATING THE INCIDENCE OF PSYCHOTIC DISORDER? ESTIMATES FROM POPULATION-BASED HEALTH ADMINISTRATIVE DATA FROM ONTARIO, CANADA

**DOI:** 10.1093/schbul/sby017.664

**Published:** 2018-04-01

**Authors:** Kelly Anderson, Ross Norman, Arlene MacDougall, Jordan Edwards, Lena Palaniyappan, Cindy Lau, Paul Kurdyak

**Affiliations:** 1 Western University; 2 Institute for Clinical Evaluative Sciences

## Abstract

**Background:**

Recent incidence estimates from population-based health administrative data in Ontario suggest an incidence rate of non-affective psychosis of 55.6 per 100,000 person-years in the general population. However, early psychosis intervention (EPI) programs across the province estimate that the treated incidence of first-episode psychosis is in the range of 12 to 13 per 100,000 per year, which corresponds to frequently cited estimates of the incidence of schizophrenia. This discrepancy between population-based estimates of incidence and the treated incidence reported by EPI programs suggests that there may be additional cases of psychotic disorder receiving services elsewhere in the health care system. Our objective was to estimate the incidence of non-affective psychosis in the catchment area of an EPI program, and compare this estimate to the EPI-treated incidence of psychotic disorder.

**Methods:**

We constructed a retrospective cohort of incident cases of non-affective psychosis in the catchment area from 1997 to 2015 using linked population-based health administrative data. Cases were identified by the presence either one hospitalization with a primary discharge diagnosis of non-affective psychosis, or two outpatient physician billings with a diagnosis of non-affective psychosis occurring within a 12-month period. We estimated cumulative incidence proportions of non-affective psychoses for the total sample meeting our case definition using denominator data obtained from the census. Using admission ratios from the EPI program (# admitted/# referred), we correct our population-based incidence estimate to yield an estimated “true incidence” of non-affective psychosis.

**Results:**

Reslts: Our case definition identified 2,864 cases of incident non-affective psychosis over the 17-year time-period. We estimate that the “true incidence” of non-affective psychosis in the program catchment area is more than twice as high as the EPI-treated incidence estimates (final numbers forthcoming).

**Discussion:**

Our findings suggest that incidence estimates obtained using case ascertainment strategies limited to specialized psychiatric services may substantially underestimate the incidence of non-affective psychotic disorders, relative to population-based estimates. We need accurate information on the epidemiology of psychotic disorders to allow service planners and administrators to more effectively resource EPI services and evaluate their coverage.

